# Venous Excess Ultrasound (VExUS Grading to Assess Perioperative Fluid Status for Noncardiac Surgeries: a Prospective Observational Pilot Study

**DOI:** 10.24908/pocus.v8i2.16792

**Published:** 2023-11-27

**Authors:** Justin C Magin, Jacob R Wrobel, Xinming An, Jacob Acton, Alexander Doyal, Shawn Jia, James C Krakowski, Jay Schoenherr, Ricardo Serrano, David Flynn, Duncan McLean, Stuart A Grant

**Affiliations:** 1 University of North Carolina School of Medicine Chapel Hill, NC USA; 2 Department of Anesthesiology, University of North Carolina Chapel Hill, NC USA

**Keywords:** perioperative care, volume assessment, POCUS, VExUS

## Abstract

**Objectives: **Perioperative fluid administration impacts the rate of complications following surgery. VExUS grading system is a standardized point of care ultrasound (POCUS)-based, comprehensive method to assess volume status. VExUS could serve as a tool to guide fluid management, if validated perioperatively. The primary aim was to assess the success rate of obtaining required windows for VExUS grading , as well as the feasibility within a perioperative setting among noncardiac surgery. Further, this study describes the incidence of perioperative venous congestion and associations with 30-day postoperative complications. **Methods: **This observational study was conducted in non-critically ill adults undergoing noncardiac surgery. Patients were scanned preoperatively, in the post anesthesia care unit (PACU), and 24 hours postoperatively for venous congestion. Researchers retrospectively captured 30-day complications for multivariate analyses. **Results: **The cohort included 69 participants. Ninety-one percent of scans over all timepoints were successfully completed. Pre-operatively, 57 (83%) scans were Grade 0, and 11 (16%) were Grade 1. Venous congestion was observed in 29 (44%) patients in the PACU (n=66). 22 (33%) patients were Grade 1, while 7 (11%) were Grade 2. At 24 hours (n=63), 31 patients (49%) had venous congestion: 20 (32%) Grade 1 and 11 (17%) Grade 2. Of the pre-operative Grade 0, 28 (50%) had at least one postoperative scan with venous congestion. No patients were Grade 3 at any timepoint. The 30-day complication rate was 32% (n=22). Eleven (16%) patients developed acute kidney injury (AKI). There was no statistically significant association between VExUS grading and all-cause complications or AKI. **Conclusion: **This study demonstrates that perioperative VExUS scoring is a feasible tool among a variety of noncardiac surgeries. We highlight that venous congestion is common and increases postoperatively within non-ICU populations. Larger studies are needed to assess the relationship between VExUS grading and postoperative complications.

## Introduction

### Developing an Understanding of Volume Status

Despite significant improvements in perioperative safety, surgery continues to carry significant risks of major morbidity and mortality. Moreover, 30-day complication rates following major abdominal surgery range between 5.8-43.5% 1.

Perioperative fluid administration can impact the rate of complications following surgery. Hypovolemia is common due to 8-hour fasts before surgery, intraoperative blood loss, and evaporative losses that occur during surgery 2. However, excessive fluid administration can lead to hypervolemia, precipitating organ dysfunction, respiratory failure, and delayed wound healing 2, 3. Implementing enhanced recovery after surgery (ERAS) pathways has been associated with improved surgical outcomes and decreased length of stay 2. Though these methods may improve care on the population level, it is unclear how often patients experience hypo- or hypervolemia following surgery. 

Current volume status assessments via physical exam, urine output, and vital signs are poor. Quantitative assessment is traditionally performed through invasive measurement via central venous or pulmonary artery catheters. Echocardiography can be used to estimate volume status, but the application of perioperative transesophageal echocardiography remains primarily limited to cardiac surgery. Furthermore, noninvasive ultrasound measurement of the inferior vena cava (IVC) collapsibility does not reliably capture hypervolemia.

### The emergence of POCUS techniques

Point of care ultrasound (POCUS) is expanding due to the decreased cost of diagnostic ultrasound machines. In critical care, heart, lung, and venous protocols have been described to identify fluid imbalances 4, 5. POCUS has improved the fluid assessment and has even influenced decisions that significantly decreased volume status and ventilator dependence 6. 

The venous excess ultrasound (VExUS) grading protocol is a standardized POCUS-based method that incorporates measurements of the IVC along with Doppler scans of the portal, hepatic, and intrarenal veins. The IVC measurement and Doppler scans correlate to minimal, moderate, severe, or no venous congestion. High VExUS scores have been associated with acute kidney injury after cardiac surgeries 7. Further, VExUS has been used in ICUs to guide clinical decision-making in patients experiencing clinical deterioration^8^ and recently showed that patients who had a reduction in VExUS score did clinically better in terms of having more renal replacement therapy-free days in an ICU population 9. However, VExUS grading has yet to be studied immediately around noncardiac surgery. VExUS ultrasound views might not be obtainable in the perioperative period following open and laparoscopic abdominal surgery because abdominal gas deteriorates ultrasound imaging. VExUS, if practical in the immediate postoperative period and validated in a clinical setting, could provide a noninvasive, individualized method to optimize fluid management in surgical patients.

We hypothesized that after losing the tight control of fluid administration in the operating room, a percentage of patients would develop volume overload over time. This study’s main aim was to evaluate if VExUS windows are possible to obtain perioperatively following abdominal surgeries and subsequently assess both venous congestion and the incidence of volume overload. Secondarily, our study assessed the risk of surgical complications with the incidence of venous congestion by VExUS grade in the pre-operative, immediate postoperative, and 24-hour postoperative time intervals. 

## Methods

We conducted a single center, prospective study enrolling non-critically ill adults 18 years and older undergoing gynecologic, thoracic, urologic, laparoscopic colorectal, or vascular surgery at the University of North Carolina Medical Center from June 1, 2022 to August 1, 2022. We obtained institutional review board (IRB) approval in April 2022 and operated under institutional guidelines. A written informed consent and HIPAA Authorization form was completed for each enrolled patient. VExUS scans were performed using a Kosmos Ultraportable Torso-One device (Echonous Inc. Bothell WA) or a Fujifilm Sonosite LX device (Fujifilm Sonosite Inc. Bothell WA). Patients with pre-surgical AKI, delirium, portal thrombus, end-stage cirrhosis, as well as those undergoing cardiac surgery or open exploratory laparotomy abdominal surgery were excluded.

Non-mechanically ventilated subjects received VExUS protocol-guided ultrasound scanning pre-operatively, postoperatively (0-6 hours after surgery, in PACU), and approximately 24 hours postoperatively (18-30 hours after surgery). An operator did a qualitative cardiac scan with a phased array probe on cardiac preset before each VExUS scan to assess for obvious right ventricle dysfunction or other major cardiac abnormalities. EKGs were not regularly completed with VExUS scans, since the study scanned amongst routine clinical care downtime. Scans were completed using the curvilinear probe or the abdominal preset depending on whether the Echonous portable probe or Sonosite was used, respectively. The operator first attempted to visualize IVC in the subxiphoid window but then would elect for midaxillary, if necessary. The IVC was viewed in long axis with special attention taken to observe widest perpendicular diameter. The hepatic, portal, and intrarenal veins were visualized in the midaxillary window. Focused effort was made to scan the main hepatic, main portal, and interlobar renal veins and not the possible other branches of each respective vessel. To obtain sufficient doppler signals, breath holding was conducted by the participant to stabilize the diaphragm and secure adequate windows. This brief breath holding was done at various points throughout the respiratory cycle depending on what would elicit the best anatomical views for using doppler. Scans were completed by medical students that have undergone thorough POCUS training through scholarly concentration and operated under the guidance of anesthesiologists. Before conducting the study, adequate institutional training, and experience on obtaining views and assessing sufficiency of doppler was performed. Although all members are thoroughly trained in ultrasound, no physicians hold unique certifications. VExUS grades were confirmed by two operators. Any discrepancy in scoring was decided by a third interpretation, the principal investigator. We collected patients’ ASA Physical classification scores^10^ and 30-day complications were observed according to ACS NSQIP definitions^11^ and measured using the ACS NSQIP risk calculator 12. Postoperative complications were confirmed by electronic medical record chart review after at least 30 days from operative date. AKI was defined according to KDIGO as SCr >0.3mg/dl from baseline within 48hr, greater than or equal to 1.5 times baseline SCr within 7 days, or urine output less than 0.5mL/kg/hr for greater than 6 hours 13. Notably, the National Institutes of Health supported funding for this study with grant number T35-DK007386. 

### Statistical Analysis

Previous pilot data around gastric ultrasound scanning identified initial success rates up to 68% and that with training scanning could reach 95% success rate 14, 15. With this in mind, we defined feasibility as postoperative scanning success of 95% or better. VExUS grades were treated as ordinal variables in the R code, and the Kruskal-Wallis rank sum test was used to test the association. Scans that were not scorable were excluded from the analyses. A P-value of 0.05 was used to determine statistical significance. This manuscript adheres to the applicable CONSORT/STROBE guidelines.

### Ethics

Ethical approval for this study (IRB number 22-0796) was provided by the Institutional Review Board Committee at the University of North Carolina, Chapel Hill, North Carolina, United States (Approved by Sherry Whittaker) on 20 May 2022.

## Results

### Demographics and Practicality

Seventy-six patients were approached for this study, of which 69 consented and enrolled. The median age of the cohort was 62 (range 19, 88). Forty-one (59%) identified as female, and 46 (67%) identified as white. The cohort’s median BMI was 28 (range 16, 44). Additional demographic data are presented in Table 1. VExUS scanning was successfully completed across all three timepoints in 91% of patients. Over the three timepoints, only 12 scans could not be scored. In the immediate postoperative period, 66 (96%) scans were successfully graded, and only three scans could not be obtained: one due to intra-abdominal gas accumulation after a laparoscopic procedure and two due to patient refusal in the setting of poorly controlled pain. At the 24-hour timepoint, 63 (91%) were successfully graded and scans were not obtained on six patients. One due to intra-abdominal gas and five due to early patient discharge. The two scans that were unable to be scored due to intra-abdominal gas were both due to inability to measure the IVC. In scans where the IVC could be measured, we were able to complete all other views successfully. Excluding the early discharges, at 24-hour timepoint, 63 (98%) of the 64 non-discharged patients were successfully scanned. The median calculated NSQIP risk score for any 30-day complication was 10.9%, along with a calculated risk for serious 30-day complications of 9.6%.

**Table 1:** Demographic and Clinical Characteristics in the Overall Study Cohort

-	**Entire Cohort **(N = 69)
Age	62 (48, 67)
Female	41 (59%)
**Race ** Black White Other	- 18 (26%) 46 (67%) 5 (7%)
BMI	28 (24, 32)
Length of Stay, days	2 (1, 4)
Median Baseline Serum Creatinine, mg/dL	0.89 (0.7, 1.1)
ASA	3 (3, 3)
NSQIP risk, mean	10.9%
NSQIP serious risk, mean	9.6%
Any 30-day complication	22 (32%)
*IQR within parentheses unless otherwise specified	

### Incidence of Volume Overload

**Figure 1:**
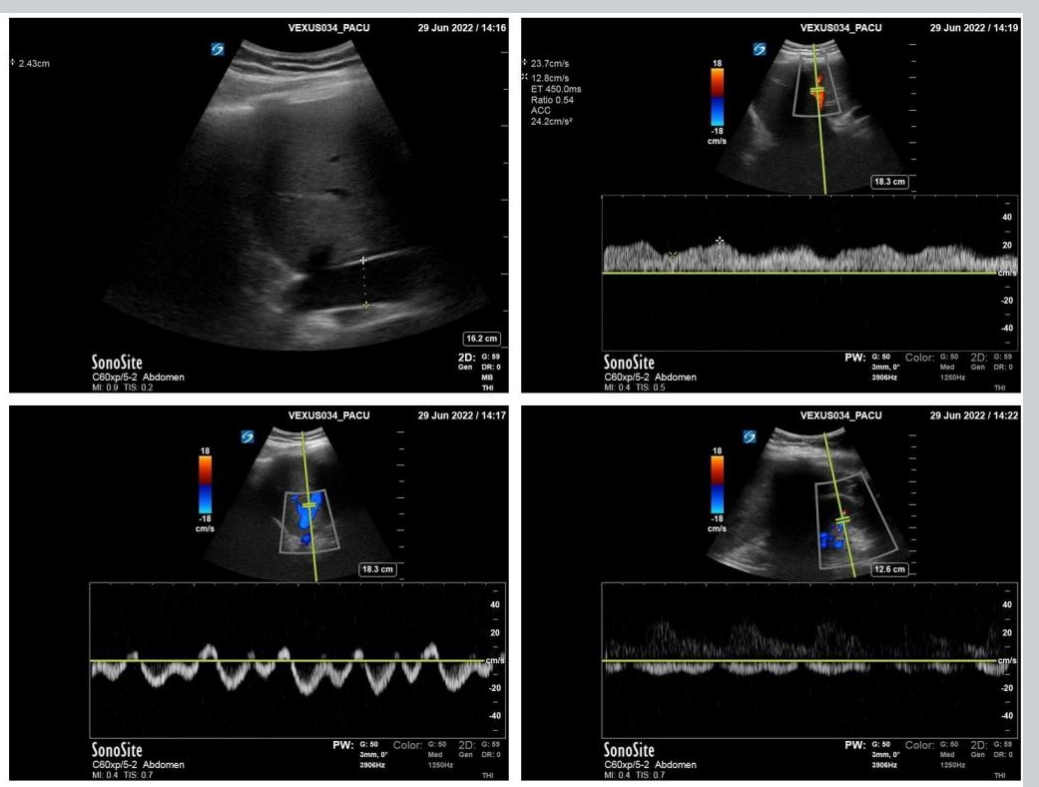
Example of a PACUVExUS scan with Grade 1: mild congestion. A) IVC measured at 2.43 cm through hepatic window. B) Doppler waveform on hepatic vein. C) Doppler waveform of portal vein with pulsatility measurement. D) Doppler waveform on intrarenal vein.

**Figure 2:**
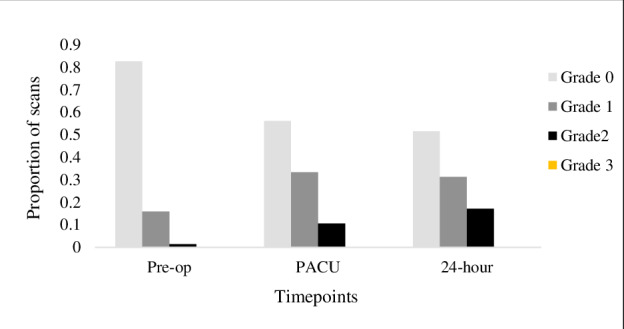
VExUS grading scores over study timepoints.

The incidence of congestion on individual scanning views is described in Supplemental Table S1-S3. Of the 69 scans obtained pre-operatively, 13 (19%) had positive VExUS grades. Of these, 12 (92%) were identified as mild congestion (Grade 1) and one as moderate (Grade 2). Of the 66 available PACU scans, positive VExUS grades were observed in 29 (44%) patients. Twenty-two (33%) had mild congestion, while 7 (11%) had moderate congestion. Figure 1 highlights an example of mild venous congestion in the PACU. Of the 63 patients that received a 24-hour scan, nearly half (31 patients) had evidence of venous congestion based on VExUS score. Of these, 20 patients had mild congestion (Grade 1) and 11 had moderate congestion (Grade 2). No patients were assessed to have VExUS Grade 3, indicating severe congestion. General VExUS grading increased over time, as shown in Figure 2. Of the 56 patients with Grade 0 before surgery, 28 (50%) had at least one scan indicating venous congestion post-operatively. The incidence of Grade 2 congestion was highest at the 24-hour timepoint (n=11, 17%), and of those, 10 (91%) were due to severe pulsatile congestion observed in the portal vein.

### Outcomes, Complications, and Statistical Analysis

Of the 69 patients, 22 (32%) had at least one 30-day complication. Eleven (16%) individuals met the criteria for postoperative AKI, and 8 (72%) of the 11 were either partial or radical nephrectomies. Other complications included 5 (7%) readmissions, 4 (6%) surgical site infections, 3 (4%) return to OR, 3 (4%) post-op hemorrhages, 1 (1%) pulmonary embolism, and 2 (3%) episodes of cardiac arrest with one resulting in death (1%). 

**Figure 3:**
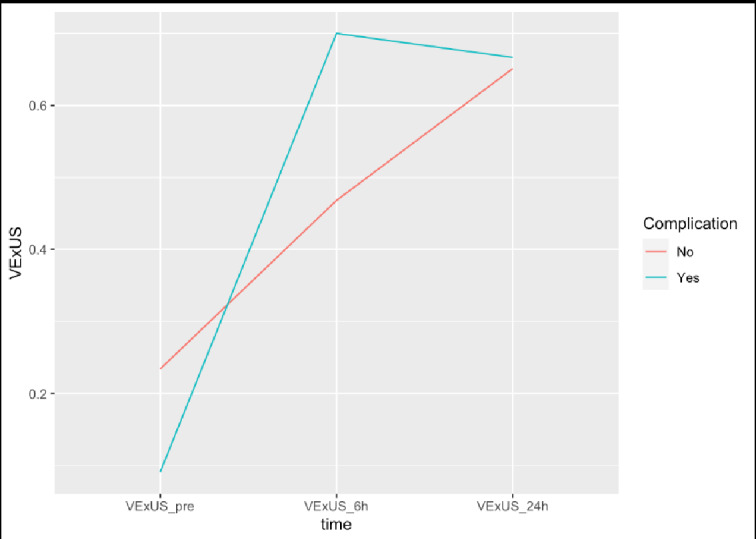
OrdinalVExUS grades over study timepoints stratified by all-cause 30-day complications cohort and no complication cohort

**Figure 4:**
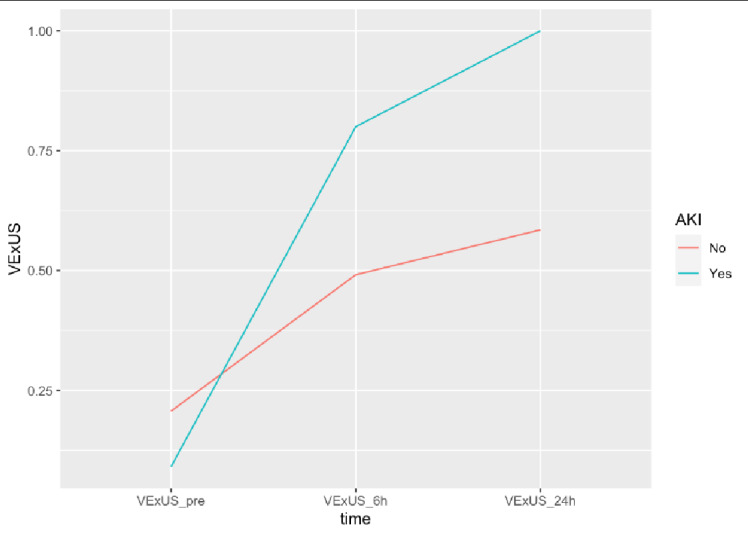
OrdinalVExUS grades over study timepoints stratified by AKI cohort and no AKI cohort

VExUS grade at the scanning period was assessed for an association with postoperative 30-day complications as well as AKI, individually. However, no statistically significant association was observed between grading and complications or AKI (Table 2-3). Moreover, no covariates (age, sex, race, LOS, ASA) had a statistically significant association with complications (Table 2-3). VExUS grading scores of those with and without complications over the three scanning timepoints are depicted in Figures 3-4.

**Table 2:** VExUS scores associations with 30-day complications

-	**No Complication** (n=47)	**Any Complication** (n=22)	**Overall** (N=69)	**P-value**
Pre-op VExUS^a^ – mean (SD)	0.23 (0.48)	0.09 (0.30)	0.19 (0.43)	0.436
PACU^b^ VExUS^a^ –mean (SD)	0.47 (0.65)	0.70 (0.73)	0.54 (0.68)	0.444
24-hr VExUS^a^ – mean (SD)	0.65 (0.75)	0.67 (0.80)	0.66 (0.76)	0.997
Age–median [IQR]	63 [50, 68]	58 [41, 66]	62 [48, 67]	0.490
**Sex** Female Male	- 31 (66%) 16 (34%)	- 10 (46%) 12 (54%)	- 41 (59%) 28 (41%)	0.271
**Race** Black White Other	- 12 (25%) 30 (64%) 5 (11%)	- 6 (27%) 16 (73%) 0 (0%)	- 18 (26%) 46 (67%) 5 (7%)	-
ASA score- mean (SD)	2.8 (0.51)	2.7 (0.70)	2.8 (0.57)	0.922
Length of Stay - mean (SD)	2.7 (2.0)	3.7 (2.8)	3.0 (2.3)	0.216
^a^VExUS- venous excess ultrasound score ^b^PACU- post-anaesthesia care unit				

**Table 3:** VExUS scores associations with AKI outcome

-	**No Complication** (n=58)	**Any Complication** (n=11)	**Overall** (N=69)	**P-value**
Pre-op VExUS – mean (SD)	0.21 (0.45)	0.09 (0.30)	0.19 (0.43)	0.715
PACU VExUS – mean (SD)	0.49 (0.66)	0.80 (0.79)	0.54 (0.68)	0.418
24-hr VExUS – mean (SD)	0.59 (0.75)	1.00 (0.78)	0.66 (0.76)	0.256
Age–median [IQR]	62 [49, 67]	62 [44, 67]	62 [48, 67]	0.961
**Sex** Female Male	- 37 (64%) 21 (36%)	- 4 (36%) 7 (64%)	- 41 (59%) 28 (41%)	0.236
**Race** Black White Other	- 15 (26%) 38 (66%) 5 (8%)	- 3 (27%) 8 (73%) 0 (0%)	- 18 (26%) 46 (67%) 5 (7%)	-
ASA- mean (SD)	2.8 (0.55)	2.6 (0.67)	2.8 (0.57)	0.708
Length of Stay - mean (SD)	2.9 (2.1)	3.6 (3.4)	3.0 (2.3)	0.688
^a^VExUS- venous excess ultrasound score ^b^PACU- post-anaesthesia care unit				

## Discussion

Our study demonstrates that the VExUS grading system can be used to assess perioperative intravascular volume status in patients undergoing abdominal surgery. Our results indicate that venous congestion is common following surgery, increasing at 24 hours compared to the immediate postoperative period. Our study did not demonstrate an association between VExUS grade and 30-day complications. 

The current literature on VExUS use is scant and mainly revolves around feasibility at the bedside in critical care settings or by nephrologists. Beaubien-Souligny et al. described the first specifically post-surgical use of VExUS in the ICU after cardiac surgeries. They demonstrated the association of higher VExUS grades with an increased risk of AKI^8^. Similar studies in intensive care settings have shown that VExUS can predict adverse kidney events and aid decision-making on whether to continue volume depletion in cardiorenal syndrome^16^, ^17^. 

It was previously uncertain whether VExUS could be used in the PACU and surgical floors so soon after a wide scope of surgeries. This study exceeded the predefined feasibility target metric of 95% completed scans postoperatively. Thus, we confirm the feasibility and utility of VExUS use by anesthesiologists in this perioperative setting for noncardiac surgery patients. We found that VExUS grading could be successfully performed on most patients pre-operatively, in the post-anesthesia care unit (PACU), and the day after surgery. Moreover, each scanning session took under 10 minutes, was well-received by patients, and did not lead to delays in the pre- or postoperative holding areas. Although not an objective aim of the study, both ultrasound devices were sufficient in quality and feasibility of obtained images. Due to the larger screen and ease in seeing smaller vessels, we did elect to use the Sonosite more towards the end of the study. Venous congestion was common, occurring in 44% of patients in the PACU and 49% of patients 24 hours after surgery. Mild and moderate venous congestion has been associated with increased kidney injury, but the true extent of this risk is unknown. These findings suggest that perioperative fluid management may be excessive in some patients, and further refinement of goal-directed fluid management protocols at our institution is warranted.

We did not observe an association between positive VExUS grades and perioperative complications. However, we studied a heterogeneous group of surgical procedures and looked at 30-day postoperative surgical complications. Moreover, we used a convenience sample for this feasibility study, which lacked the power to detect meaningful differences. A prospective study sufficiently powered that would intervene when positive VExUS scans were discovered requires hundreds of patients if the complication rate was similar to our 30%. Also, at this stage the definitive effective interventions after positive VExUS scans following abdominal surgery are not yet understood. A prospective randomized intervention study would be important because of the large number of major abdominal surgeries performed each year. The results presented here do fall in line similarly with a 150 subject, prospective general ICU cohort that also did not see associations with AKI or month long outcomes^18^. This was also probably underpowered for this outcome.

Our study is limited by the narrow study window. Further, our secondary complication outcomes are limited by relatively small sample size for such generalizable outcomes. Moreover, due to the potential ambiguity of Doppler scanning results, there is a possibility for bias in the grading based on other clinical presentations at the time of scanning. However, we mitigated this risk by using two individuals for scanning who came to a consensus on all grades.

Limitations of the VExUS scans are present at various parts of the protocol. For instance, hepatic vein flow can be affected by tricuspid insufficiency 19, while intrarenal vein waveform can be challenging to capture and is the most common reason for failed scoring. Moreover, although we were attuned to common pitfalls with hepatic vein interpretations, since we did not use ECG tracing with hepatic veins there could be episodes of error with interpretation of doppler wave patterns thus introducing further limitations to this study. Although the qualitative cardiac views would have detected significant right ventricle (RV) or tricuspid dysfunction, we cannot be certain that no patients had tricuspid or RV pathology that may have impacted VExUS grading. Portal vein pulsatility can be seen without underlying pathology in an inverse relationship to body mass and thus could cause higher skewed scoring to occur^20^. At the 24-hour timepoint, 91% of the Grade 2 scores were due to portal vein pulsatility. Yet, the portal vein pulsatility could be due to iatrogenic causes such as increased abdominal pressure from laparoscopic procedures causing pulsatile mimicry or unnoticed interference from the hepatic artery.

## Conclusion

This study demonstrates that the VExUS protocol is feasible and can be performed by anesthesiologists to assess venous congestion in noncardiac surgery during the perioperative period. Further, this study demonstrates that venous congestion increases following abdominal surgery and venous congestion is a problem that should be considered beyond cardiac surgery or in an ICU. In the future, larger studies should be performed to evaluate the impact of venous congestion on perioperative complications. Additional research should be conducted to determine if VExUS can be used not only as a diagnostic tool but for patient intervention in the perioperative period to target fluid administration or diuresis.

## Disclosures

The authors report no conflicts of interest related to this work.

## Supplementary Material

Supplemental Tables S1-S3
