# Supplementary material for: Venous Excess Ultrasound (VExUS Grading to Assess Perioperative Fluid Status for Noncardiac Surgeries: a Prospective Observational Pilot Study
Source: POCUS J. 2023 Nov 27;8(2):223–9. doi: 10.24908/pocus.v8i2.16792 (PMC10721303; doi:10.24908/pocus.v8i2.16792)
Supplement: Supplemental Tables S1-S3 [file pocusj-08-16792-s001.pdf]

SUPPLEMENTAL TABLES

Table S1: View-specific Preoperative Timepoint (N=69)

	N/A[a]	Normal	Mild	Severe
Hepatic vein	58	8	3	0
Portal vein	58	6	4	1
Intrarenal vein	58	6	5	0

[a] N/A attributed when IVC<2cm and if doppler was unable to detect the signal with an appropriate view.

Table S2: View-specific PACU Timepoint (N=66)

	N/A[b]	Normal	Mild	Severe
Hepatic vein	36	25	5	0
Portal vein	36	12	11	7
Intrarenal vein	37	23	6	0

[b]N/A attributed when IVC<2cm and if doppler was unable to detect the signal with an appropriate view.

Table S3: View-specific 24-hour Timepoint (N=63)

	N/A[a]	Normal	Mild	Severe
Hepatic vein	32	29	1	1
Portal vein	32	16	5	10
Intrarenal vein	32	25	6	0

[a]N/A attributed when IVC<2cm and if the Doppler was unable to detect the signal with an appropriate view.
